# Comparing a paper based monitoring and evaluation system to a mHealth system to support the national community health worker programme, South Africa: an evaluation

**DOI:** 10.1186/1472-6947-14-69

**Published:** 2014-08-09

**Authors:** Sunisha Neupane, Willem Odendaal, Irwin Friedman, Waasila Jassat, Helen Schneider, Tanya Doherty

**Affiliations:** 1 School of Public Health, University of the Western Cape, Cape Town, South Africa; 2 Health Systems Research Unit, Medical Research Council of South Africa, Cape Town, South Africa; 3 Seed Trust, Durban, South Africa; 4 Health Systems Trust, Durban, South Africa

**Keywords:** Community health workers, Monitoring and evaluation, mHealth, Community based services

## Abstract

**Background:**

In an attempt to address a complex disease burden, including improving progress towards MDGs 4 and 5, South Africa recently introduced a re-engineered Primary Health Care (PHC) strategy, which has led to the development of a national community health worker (CHW) programme. The present study explored the development of a cell phone-based and paper-based monitoring and evaluation (M&E) system to support the work of the CHWs.

**Methods:**

One sub-district in the North West province was identified for the evaluation. One outreach team comprising ten CHWs maintained both the paper forms and mHealth system to record household data on community-based services. A comparative analysis was done to calculate the correspondence between the paper and phone records. A focus group discussion was conducted with the CHWs. Clinical referrals, data accuracy and supervised visits were compared and analysed for the paper and phone systems.

**Results:**

Compared to the mHealth system where data accuracy was assured, 40% of the CHWs showed a consistently high level (>90% correspondence) of data transfer accuracy on paper. Overall, there was an improvement over time, and by the fifth month, all CHWs achieved a correspondence of 90% or above between phone and paper data. The most common error that occurred was summing the total number of visits and/or activities across the five household activity indicators. Few supervised home visits were recorded in either system and there was no evidence of the team leader following up on the automatic notifications received on their cell phones.

**Conclusions:**

The evaluation emphasizes the need for regular supervision for both systems and rigorous and ongoing assessments of data quality for the paper system. Formalization of a mHealth M&E system for PHC outreach teams delivering community based services could offer greater accuracy of M&E and enhance supervision systems for CHWs.

## Background

Community health workers (CHWs) play an important role globally; not only in improving the health status of remote communities but also in influencing social determinants and policies which affect the overall development of the community [1,2]. CHWs provide preventive health services, improve access to basic health care services [3], collect health related data [4], monitor the community’s health [5], and act as an interface between the community and the health system [6]. CHWs are also increasingly treated as a formal part of national health systems and as a recognized delivery platform [7,8]. In recent years their role has expanded to include follow up of people with chronic conditions (HIV and other chronic diseases), administration of treatment as part of integrated community case management (iCCM) of malaria, pneumonia and diarrhoea and in some countries treatment of neonatal sepsis [7-10].

A study by the World Health Organization (WHO) and Aga Khan University [11] published in 2011 highlights and supports other studies emphasizing the importance of integrating CHWs into the primary health care delivery system especially to achieve Millennium Development Goals (MDGs) 4 and 5 [8,11-14]. South Africa is a country that has shown little progress in meeting MDG4 with an annual rate of under five mortality reduction of only 1.4% [15]. Also, MDG5 still stands at 300 maternal deaths per 100,000 live births [16]. In an attempt to improve progress towards MDG 4 and 5 as well as other disease burdens, South Africa recently introduced a re-engineered Primary Health Care (PHC) strategy, which has led to the development of a national CHW programme. CHWs are now organized in municipal ward-based PHC outreach teams of six to ten CHWs, led by a professional nurse. These teams are tasked with providing home-based health promotion activities, primarily focusing on four client types: pregnant and postnatal women, children under five and individuals requiring treatment adherence support for chronic illnesses.

While considering the integration of CHW programs at national level, studies have demonstrated that monitoring and evaluation (M&E), supervision and accountability remain key elements to ensure the effectiveness of CHW services [10,17-19]. Traditionally, CHWs use paper-based reporting forms that are regularly submitted to their supervisors. The challenges of a paper-based M&E system include issues such as an inefficient filing system; lack of supervision of record keeping; data loss; storage space; time consuming; and difficulty in tracing referrals made by the CHWs [20-23]. Paper forms are also cumbersome to carry and clients are often concerned about the confidentiality of the information captured [24]. Mobile communication technologies (referred to as mHealth) may eliminate some of these challenges. mHealth is already used for health promotion, education and awareness, adherence to chronic medication [25,26], the collection of surveillance data [23], and also in community-based CHW programmes [27,28].

Implementing community-based services using PHC outreach teams require a sound M&E system to record the strengths and weaknesses of the programme and to track progress of the programme in improving health outcomes. There are few studies that compare the traditional paper-based M&E system with a mHealth system in the context of a national CHW programme [29-32]. The aim of this paper is to contribute in this knowledge gap and provide insights on the application of mHealth as an M&E system, and how that compares to the traditional paper-based M&E system. In addition, the paper explores the practicality, accuracy and supervisory capabilities of the phone-based M&E system compared to that of the traditional paper based M&E system to support the work of CHWs. It aims to contribute towards understanding the potential impact of mHealth on service delivery processes and identifying the challenges for scaling up and sustaining a mHealth M&E system for a national CHW programme.

## Methods

### Study site

The evaluation was conducted in the Greater Taung municipality of the Dr. Ruth Segomotsi Mompati (RSM) district of the North West Province (NW), one of the poorest of the nine South African provinces. It was undertaken with one PHC outreach team consisting of ten CHWs and one team leader (a nurse). The CHWs did not have professional training and were recruited locally. The Greater Taung municipality has a population density of 32 people per km^2^ and is divided into 26 municipal wards [33]. This area is a remote deprived rural setting with 43% poor households (highest percentage of poor households among the municipalities of the RSM district) [34].

### The M&E systems

Training of the CHWs on paper M&E forms (Additional file 1) started in the beginning of 2012 and training on the phone system started in August 2012 with the same group of CHWs. The data in this paper includes the activities recorded by the CHWs between September 2012 and January 2013. The forms and mHealth survey system were developed in accordance with the PHC reporting requirements of the District Health Information System (DHIS) of South Africa. Continuous support and guidance was provided to the CHWs during site visits over the evaluation period.

#### *Paper-based M&E system*

The CHWs recorded data on various health indicators (Table 1) during daily client visits on the *CHW household visit tick sheet* and summarized this information onto the *CHW household visit weekly summary sheet.* At the end of the month, they transferred their weekly summaries onto a *CHW household visit monthly summary sheet* (Additional file 1) and submitted it to the team leader. The team leader aggregated each CHW’s monthly data onto the *PHC Outreach team monthly activity summary form*, and also prepared an *Outreach team final monthly report* for submission to the PHC facility managers. During client visits, CHWs had an option to refer clients to the health facility using a *Referral form.* Clients were encouraged by the CHWs to follow up with their referral to the clinics within 14 days. If the referred client arrived at the facility within the recommended 14 days, the facility manager captured the outcome on the rear side of the paper referral form (hence called back referral form) and handed it to the client. CHWs would then collect the back referral form from the client and record the outcome and follow up with the client if necessary.

**Table 1:** Indicators: daily client visit activities

	**Indicators**
Household activity*	Pregnancy visits
	Postnatal visits
	Children under 5 supported**
	Treatment adherence support***
	Home based care provided^++^
Referrals and supervision	Clinic referral forms issued
	Supervised visits
	Follow-up visits

The types of visits used as indicators in the study to measure correspondence, supervision capability and comparing the paper and phone data collection systems.

*Household activities refer to the services or activities rendered at the household by a CHW regarding pregnancy, PN, children under 5, adherence support and home based care. CHWs are asked to tick each box one time per household if they receive mentioned services.

**Under 5: Ticked if any children under the age of five have received services by CHW during the household visit.

***Treatment adherence support: Ticked if at least one person of the household is taking chronic medication and received adherence support from CHW during the household visit.

++Home based care: Ticked if support was provided by the CHW for someone with a disability or who is unable or needs help to complete the activities of daily living during the household visit eg., home nursing, palliative care.

#### *Phone-based M&E system*

The CHWs were given Nokia C5-00 phones to record exactly the same information as in the paper forms (Additional file 2). The participating healthcare facilities were issued with similar phones for the referral process. Automated weekly and monthly reports per CHW and aggregated for the team were generated through a software program called Mobenzi Outreach. The team leader was issued with a Samsung Galaxy tablet, and through a web-based console she could view the CHWs’ work in real time. The district and facility managers had access to the console and could review the CHWs’ work in the same way as the team leader.

Regarding the referral functionality (Additional file 3), the CHW sent a referral text message to the facility for her client, and once the client accessed the service, the facility manager sent a referral response back to the CHW via the mobile phone, along with the paper (back referral form). The phone system provided a mechanism to track the referral of patients to the health facility and vice versa. If clients missed their referral dates, the respective CHWs were sent an update showing a “missed visit” on their phone so that they could follow up on their client and reschedule an appointment if needed.

### Supervision

One of the foci of this evaluation was to compare the phone and paper systems regarding supervision of CHWs. For the purpose of this evaluation, supervision is defined as reviewing CHWs’ record keeping on their clients to check if any support is needed, and supervisory visits with the CHWs to improve the quality of care to the clients.

The supervisory role of the team leader had been newly introduced with formal training in August 2012. Automated notifications were sent to the team leader’s email when the National CHW protocol was violated, for instance, when a scheduled postnatal visit was missed by a CHW, the team leader received a notification providing an opportunity to intervene. Supervisory intervention was assessed based on log-ins onto the Mobenzi system.

### Data collection

A mixed method approach was used; both qualitative and quantitative data were collected. The qualitative data were collected during a focus group discussion (FGD) with the ten CHWs towards the end of the evaluation. It focused on their experiences using the two systems, the practicality of the systems and supervision experiences. The paper-based quantitative data were collected from all of the aforementioned forms maintained by the CHWs and the team leader, and the mHealth data were accessed through the Mobenzi web-based console. The data from both systems were tabulated and analysed in Excel. The implementation and evaluation of the paper-based and cell-phone based M&E systems were approved by the North West Province Department of Health and the RSM district Management team. CHWs gave verbal informed consent for participation in the focus group discussion.

### Data analysis

The quantitative data was analysed by firstly assessing the accuracy with which the CHWs transferred the indicators (see Table 1) from their weekly visit summary sheet onto the monthly visit summary sheet. For each CHW, transfer accuracy between the monthly and weekly summaries was calculated by subtracting the weekly total from the monthly total, expressed as the percentage of correspondence. A one tailed t test (alpha = 0.05) was done to compare the improvement in correspondence over time, with September 2012 as the baseline month. Secondly, a comparative analysis (a one tailed t test with alpha = 0.05) was done to calculate the correspondence between the paper and phone records for each CHW. This was done in a similar way as for the transfer accuracy, i.e. subtracting the ‘paper total’ from that of the mHealth data total, expressed as the percentage of correspondence. A comparison was also done on the client follow-up visit data, referral data and supervised visits for both paper and phone systems. The FGD was audio-recorded and analyzed using thematic content analysis.

## Results

The average size of the CHWs’ catchment population was 146 households per CHW, ranging between 86-237 households per CHW. They supported ± 1,400 clients, ranging between 150-200 active clients per CHW (around 1.2 clients per household). Across the ten CHWs, 829 (range: 665-1175) visits were recorded per month, averaging 74.5 (range: 5-220) visits every month and approximately 3.5 visits per day per CHW.

The CHWs, during the training sessions and FGD, mentioned that they find it difficult to carry piles of paper, especially because there was no means of transportation to reach households except walking. During the FGD, when they were asked which system they thought is more conducive for building relationships with the clients, mixed responses were received: “*Paper forms, so that we can write and discuss”* ……*“Both paper and phone”……… “The phone system, because it is more confidential and the clients feel comfortable with that*.” (FGD November 2012).

The key findings have been separated into the three categories below.

### Data transfer accuracy in the paper based system

**Figure 1:**
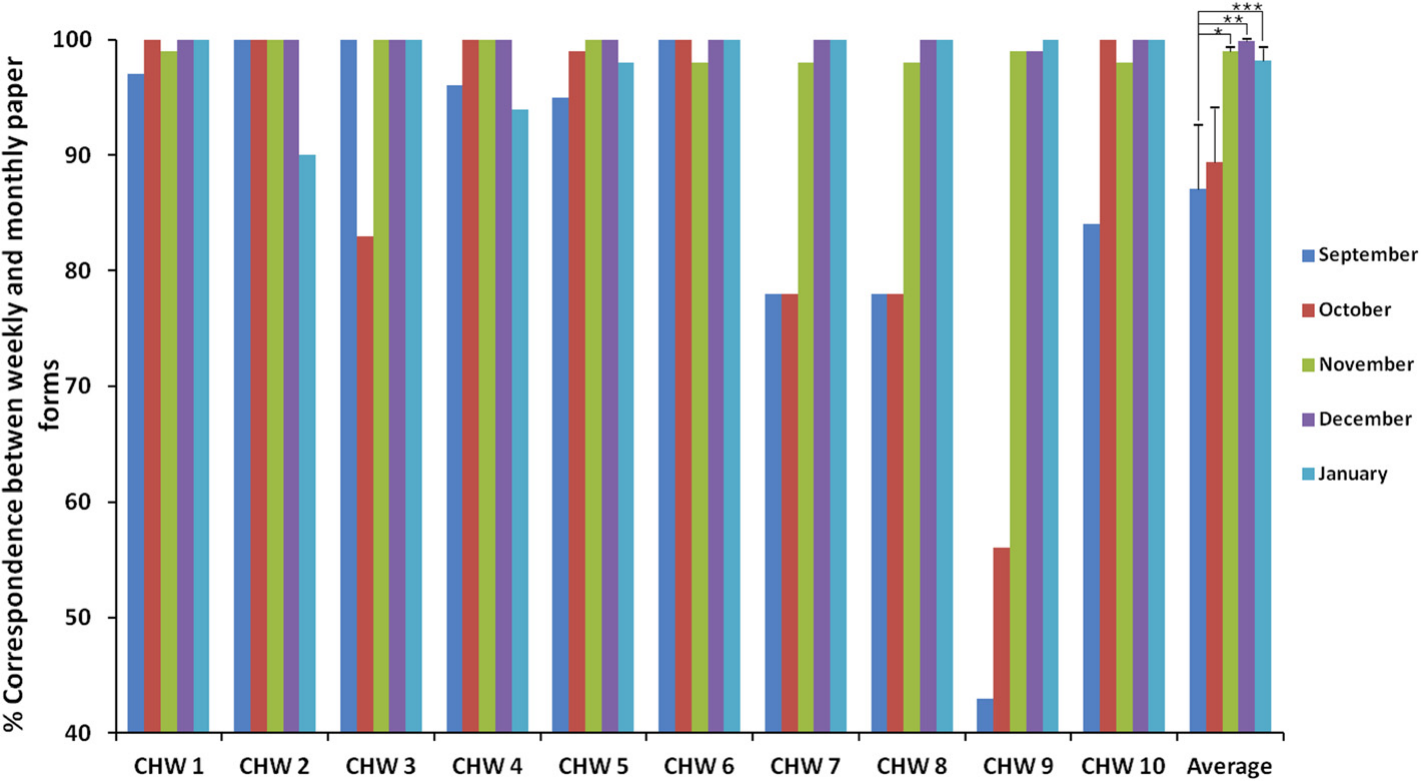
**Data transfer accuracy.** Data transfer accuracy between weekly and monthly paper forms using the ‘Follow up visits’ indicator. *indicates statistical significance (p < 0.05).

With regard to transfer from weekly to monthly forms, 40% of the CHWs (CHWs 1, 4, 5, 6), showed a consistently high level of transfer accuracy; a notable improvement over time was observed for CHWs 7 – 10 (Figure 1). CHWs 2 and 3 each had one month of poor accuracy. Overall there was an improvement over time, and by January 2013, all CHWs achieved a correspondence of 90% or above. The improvement in correspondence, compared to September, was not statistically significant for the month of October (p = 0.38), but was significant for rest of the months (Nov: p = 0.02; Dec p = 0.02; Jan: p = 0.03). The most common error that occurred was in wrongly summing the total number of visits across the five *Household activity indicators* (Table 1).

**Figure 2:**
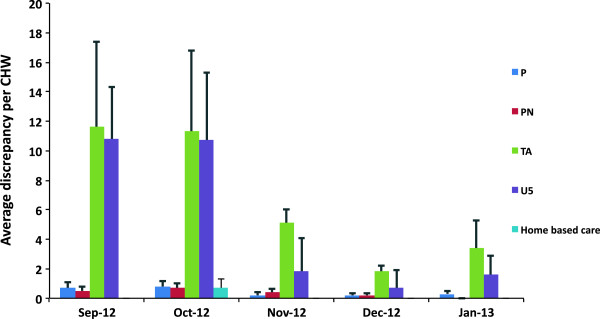
**Absolute discrepancies on paper forms with standard error of mean.** Discrepancies (average of ten CHWs) between weekly and monthly paper forms on five types of visits- Pregnancy (P), Post natal (PN), Treatment Adherence (TA), Under 5 and Home based care (U5).

A discrepancy was defined as a difference between the accumulated weekly data versus the monthly data. A further analysis of data transfer accuracy (Figure 2) showed that it was in particular for the “under 5” and “treatment adherence support” indicators, that discrepancies between the weekly and monthly totals were observed. For example, CHW1 had 68 visits on her weekly forms for “treatment adherence support”, whereas she reported 46 on the monthly form. This shows an absolute discrepancy of 22. When the CHWs were asked about the discrepancies in the paper forms, they reported that it was due to the lack of understanding the indicator definitions: *“We never got [proper] training for the paper system. We don’t know about the new paper forms. We were given the paper forms and its up to us to understand those with our knowledge. We are confused on household visits and other elements. When new tools are added, we never get additional training”* (FGD November 2012).

**Figure 3:**
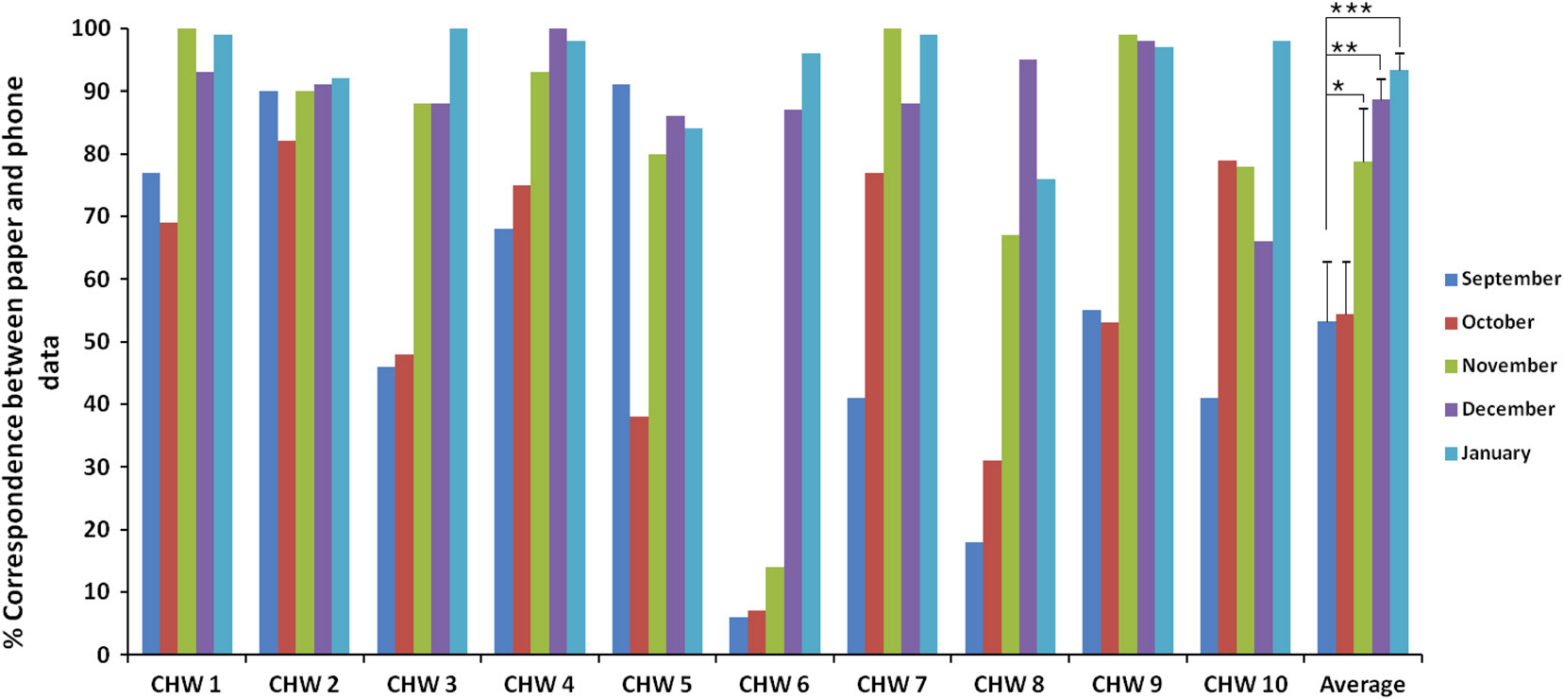
**Correspondence between paper and phone data.** Data correspondence between the paper and the phone system using the ‘Follow up visits’ indicator. *indicates statistical significance (p < 0.05).

There was a considerable reduction in discrepancies between the paper and phone data over time (Figure 3). There was no statistically significant difference for the month of October (p = 0.42), however, the improvement compared to September, was significant for rest of the months (Nov: p = 0.02; Dec: p = 0.001; Jan: p = 0.0002). The CHWs said that the reduction in discrepancies came with experience in using the systems and the regular support provided: *“Now I understand it is important to make good correspondence between phone and paper data. Comparing paper and phone data, I learnt that I need to record everything”* (FGD November 2012).

### Referral and back referral

**Figure 4:**
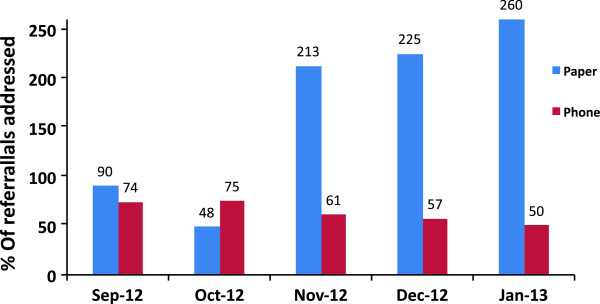
**Clinical referrals and back referrals.** Proportion of the referrals addressed and captured on the phone and the paper system. Referrals addressed on the paper system exceeds 100% due to accumulation of previous months` referrals, which were brought into the clinics in the successive month.

The proportion of referrals to the health facilities that were completed using phone and paper records respectively is illustrated in Figure 4. In the last three months, the paper data shows that more back referrals were received than referrals made. This was due to accumulation of previous months’ referrals, which were not brought in to the clinics in the same month (indicated from the mHealth data). The phone system was able to link each individual referral to its outcome, whilst in the paper system, the total count of forms sent to the clinic and forms returned to the CHW was done but these forms were not linked. This resulted in referral completion rates of over 100% on paper data (Figure 4).

The Mobenzi system provided data on the nature of the referrals made and presented a better overview on the types of health problems (Additional file 3). For instance, the most common CHW referrals were for immunizations, vitamin A supplementation and deworming. Although the referral - reasons were also recorded on paper, it was tedious and time consuming for the team leader to extract data and get an overall picture on types of referrals. The phone system was more efficient and timesaving in this regard.

### Supervision of the CHWs

The Mobenzi system allowed the team leader to log onto the web-console any time she wished to. It also sent short message service (SMS) notifications to her when visits were scheduled outside of the protocol and the number of these notifications were between 28 and 58 per month. The web-console allowed the supervisor to instantly review the information captured by the CHWs and enabled her to provide feedback to them. In addition it also kept a log of the number of times the supervisor logged into the system. During the evaluation period she logged on average twice a week into the system. There were very few supervised visits (Table 2), and there was no evidence that she followed up on the automatic SMS notifications received.

**Table 2:** Supervised visits

**Months**	**Total number of visits by 10 CHWs**	**% visits supervised by the team leader (recorded on the phone)**	**% visits supervised by the team leader (recorded on paper forms)**
Sep-12	461	1.3%	5.2%
Oct-12	664	3.2%	0
Nov-12	640	2.3%	0.6%
Dec-12	854	0.8%	0
Jan-13	1171	0.3%	0

Percentage of CHWs’ client visits that were supervised by the team leader.

In addition, the number of supervisory visits did not match in the two systems (Table 2), because the CHWs did not understand the indicator “supervised visit”: *“We may [have] made mistakes for [data indicators such as]‘campaigns’ and ‘supervised visits’* ”(FGD November 2012).

Responses from the focus group discussion confirmed the infrequent supervision: “*We would like to get together [with the team leader and other CHWs] and get a chance to learn and discuss what we are not sure about. But we do not have that opportunity. Once we are given the data collection forms, we need to understand those forms ourselves”* (FGD November 2012).

## Discussion

This evaluation demonstrates poor data transfer accuracy on the paper forms; a problem that was improved by collecting and aggregating data automatically via the mHealth system. The mHealth system, but not the paper system, enabled a longitudinal follow up of each referral so that the back referral could be linked to the original referral and subsequent referrals to the same client. A lack of supervision was observed on both systems. There was a significant improvement in data correspondence (and data accuracy) over time denoting that proper training and support makes a positive impact on CHWs data collection and reporting.

### Data transfer accuracy in the M&E systems

The integrity of CHWs’ data is critical in assessing their performance. It was observed that basic adding up and transferring from the weekly to the monthly paper forms was challenging, but not surprising since the CHWs had varying levels of education and not all have completed high school. Data accuracy was also impeded as the CHWs struggled to keep up with the changes made to the M&E forms. With the mHealth’s automated calculation functionalities, neither the CHWs nor the supervisor had to do any manual data transfer or calculations which eliminated human error. Thus, CHWs can produce accurate data, better record keeping and also use mHealth to save time to provide quality care [4,35,36].

### Practicality regarding referral forms

Although the phone and paper forms captured the same information, there was, however, a difference between the two systems. The paper forms captured the total number of referral forms given for a month and the total number of back referrals received by a CHW for all of her clients. The paper forms were however not linked to the antecedent or subsequent referrals of a client as in the phone system providing inconclusive data. In addition, clients’ health information from a rural area can get incorporated into a broader health system database (such as the DHIS) via the mHealth system [36]. In spite of the observed benefits of referrals via the phone system, it is important to note that paper referrals given to the clients might play an important role in reminding them of their clinic appointments.

The decrease in the completion of phone-based back referrals from the health facilities could be because of insufficient training of facility staff and a lack of their familiarity with the phone system. There were also no mandatory rules in placed from the district office to actively use the provided phone system.

### Supervision

Sachs et al. [8] have underscored the importance of investment in simple but excellent supervision approaches to create a high performing CHW system. The mHealth system has the potential to provide better support and supervision of the CHWs. For instance, the supervisor could regularly log in and review the CHWs’ record keeping in order to provide the necessary support; the team leader could also check for urgent problems, data accuracy or complicated cases. With the paper system, it is difficult to review the CHWs’ visits and data on a daily basis; the team leader could oversee the data collected and intervene only when the CHWs submit their forms. It is important to note that mHealth fulfilled this need of immediacy. Data such as population catchment, average household visits per day, and reasons for clinical referrals were easily traceable from the web-based console.

One of the aims of this evaluation was to explore the potential of enhanced supervision using the mHealth system. It was postulated that having daily access to real time data would enable the supervisor to identify and act on problems, such as missed visits. However, the results demonstrate that the supervision functionality of the mHealth system was under-utilised by the team leader and almost non-existent for three of the five months, and a similar pattern was observed for the paper system. This could be because supervision was new to the team leader or deemed unnecessary, lack of adequate understanding and training or simply because the team leader was over burdened with her responsibilities.

The discrepancy in data decreased and precision increased significantly on reported data over time, which may be due to an increased familiarity with the data capturing system. But importantly, CHWs had the researchers’ support and were aware that their forms were being checked for accuracy, which may have sufficed for the needed supervision. Assessing the quality of care, however, requires observing the field visits, regardless of the system used.

The potential of knowledge transfer and peer supervision among the CHWs was also observed. A few CHWs were performing better than others and could potentially have mentored those who were struggling.

### Challenges

#### *Challenges observed with the paper system*

Similar to findings in the literature [20-24], the results indicate that the CHWs have struggled with the paper M&E, irrespective of the introduction of the phones. Data transfer from weekly to monthly forms demonstrated errors on the monthly reports and minimal supervision was provided to address the issue. However, our results indicate that a paper-based system can produce accurate data if proper supervision and support is provided to the CHWs. The CHWs repeatedly mentioned that the paper forms were cumbersome to carry especially when there was no choice but to travel by foot. Moreover, the CHWs reported that clients were concerned about confidentiality of the paper forms and therefore reluctant to raise their health queries. A study done by Shozi, et al. in South Africa identified similar challenge of perceived lack of confidentiality on the paper forms [24].

#### *Challenges observed with the phone system*

There were continuous improvements to make the system more accurate, user friendly and adaptive towards the DHIS. Functionalities were being tested and added during the pilot period, which may have been challenging for the CHWs to keep up with the changes. Importantly, most of the challenges were not related to technology issues (such as not having reception on their phones when they were out in the field), but related to health systems issues: mobilising the necessary funding, reorienting training systems towards supporting mHealth and creating the capacity for ongoing technical support.

## Conclusions

Many countries in Africa are scaling up CHW programmes in order to reach the goal of universal access to health care [8]. Our evaluation has highlighted challenges with both paper and mHealth CHW M&E systems and emphasized the need for regular supervision and rigorous and on-going assessments of data quality. Real-time data availability offered by the mHealth system plays an important role in closing the gap between clients and health service providers and enables accurate tracking of referrals. It is important that mHealth is not seen as a universal panacea for problems with information systems. The challenges of scaling up a new program are not insignificant. However, this evaluation suggests that formalization of a mHealth M&E system for PHC outreach teams delivering community based services could offer greater accuracy of M&E and enhance supervision of CHWs.

## Competing interests

The authors report no competing interests.

## Authors’ contributions

SN wrote the manuscript. SN, WO and IF collected the data. SN, WO and TD contributed to data analysis and revised the manuscript. All authors reviewed and approved the final manuscript.

## Pre-publication history

The pre-publication history for this paper can be accessed here:

http://www.biomedcentral.com/1472-6947/14/69/prepub

## Supplementary Material

Additional file 1 Paper M&E forms used by the CHWs to record their household visits and monthly summary. Click here for file

Additional file 2 Mobenzi web console with CHWs household visits monthly summary captured by the phone system. Click here for file

Additional file 3 Screen shots showing mHealth referral system and referral data compiled on the web consol. Click here for file
