# Supplementary material for: Comparing a paper based monitoring and evaluation system to a mHealth system to support the national community health worker programme, South Africa: an evaluation
Source: BMC Med Inform Decis Mak. 2014 Aug 9;14:69. doi: 10.1186/1472-6947-14-69 (PMC4150556; doi:10.1186/1472-6947-14-69)
Supplement: Additional file 1 — Paper M&E forms used by the CHWs to record their household visits and monthly summary. [file 1472-6947-14-69-S1.pdf]

CHW Household Visit Tick Sheet

health
Department: Health
REPUBLIC OF SOUTH AFRICA

CHW name		Month/Year	Page no.
Clinic *(DHIS name)*	Ward *(DHIS #)*	Team *(DHIS name)*	

**Household visit details** *(note: tick only ONE type of visit)*						**Household activity** *(note: tick only once per activity)*					**Number *Referral Forms* given** *(total)*			**Activity head count** *(total)*	
			Type of visit												
Visit no.	Visit date *(dd/mm)*	Official household registration number[1]	Household registration visit	Follow-up visit	Supervised visit	Pregnancy	Postnatal	Under 5	Adherence support	Home-based care	Clinic *Referral Forms* issued	Social services *Referral Forms* issued	Home-based care *Referral Forms* issued	Clients under 5 years given service	Clients 5 years and older given service
1															
2															
3															
4															
5															
6															
7															
8															
9															
10															
11															
12															
13															
14															
15															
16															
17															
18															
19															
20															
21															
22															
23															
24															
25															
**Total**															
**Total number of support groups facilitated this week**				**Notes:**											

CHW signature ________________________ (date) _______________ Verified by TL _________________________ (date)____________

[1] Use the CHW HH identifier number if an Official HH registration number has not yet been assigned to the household

Version 1.2

August 2012

CHW Household Visit Monthly Summary Form

health
Department: Health
REPUBLIC OF SOUTH AFRICA

CHW name	Reporting month/year
Clinic *(DHIS name)*	Ward *(DHIS #)*
Team *(DHIS name)*	

Household visit details *(totals)*					Household activity *(totals)*					***Referral Forms*** **given** *(totals)*			Activity head count *(totals)*		Support groups facilitated *(total)*
		Type of visit													
Tick sheet no.	Tick sheet end date *(dd/mm)*	Household registration visit	Follow-up visit	Supervised visit	Pregnancy	Postnatal	Under 5	Adherence support	Home-based care	Clinic	Social Services	Home-based care	Clients UNDER 5 years	Clients 5 years and older	
1															
2															
3															
4															
5															
6															
7															
8															
9															
10															
**Monthly Total**															
**Total community campaigns this month**															

CHW signature ______________________ Date ______________

Verified by Team Leader ______________________ on ______________ (date)

Version 1.2
August 2012
