# Supplementary material for: Comparing a paper based monitoring and evaluation system to a mHealth system to support the national community health worker programme, South Africa: an evaluation
Source: BMC Med Inform Decis Mak. 2014 Aug 9;14:69. doi: 10.1186/1472-6947-14-69 (PMC4150556; doi:10.1186/1472-6947-14-69)
Supplement: Additional file 2 — Mobenzi web console with CHWs household visits monthly summary captured by the phone system. [file 1472-6947-14-69-S2.pdf]

CHW Household Visit Monthly Summary

Month: Feb 2014 | CHW Name: _ | Type: Monthly Summary | Display

Household visit details (totals)					Household activity (totals)					Referral Forms given (totals)			Activity head count (totals)	
		Type of visit												
Tick sheet no.	Tick sheet end date (dd/mm)	Household registration visit	Follow-up visit	Supervised visit	Pregnancy	Postnatal	Under 5	Adherence support	Home-based care	Clinic	Social services	Home-based care	Clients UNDER 5 years	Clients 5 years and older
1	10/02	0	25	0	2	0	17	16	5	0	0	0	19	17
2	12/02	0	25	0	1	1	19	13	1	0	0	0	26	13
3	18/02	0	25	0	0	0	17	13	1	0	0	0	23	10
4	26/02	0	25	0	2	3	17	11	3	0	0	0	25	15
5	27/02	0	5	0	2	1	2	3	0	0	0	0	5	7
6														
7														
8														
9														
10														
Monthly Total		0	105	0	7	5	72	56	10	0	0	0	98	62

Total community campaigns this month [ ] Save

Total number of support groups facilitated this month [ ] Save

Generate PDF
