# Supplementary material for: Comparing a paper based monitoring and evaluation system to a mHealth system to support the national community health worker programme, South Africa: an evaluation
Source: BMC Med Inform Decis Mak. 2014 Aug 9;14:69. doi: 10.1186/1472-6947-14-69 (PMC4150556; doi:10.1186/1472-6947-14-69)
Supplement: Additional file 3 — Screen shots showing mHealth referral system and referral data compiled on the web consol. [file 1472-6947-14-69-S3.pdf]

Screen Shot 1: mHealth-based referral system
Screen Shot 2: Referral data from the web consol

1.

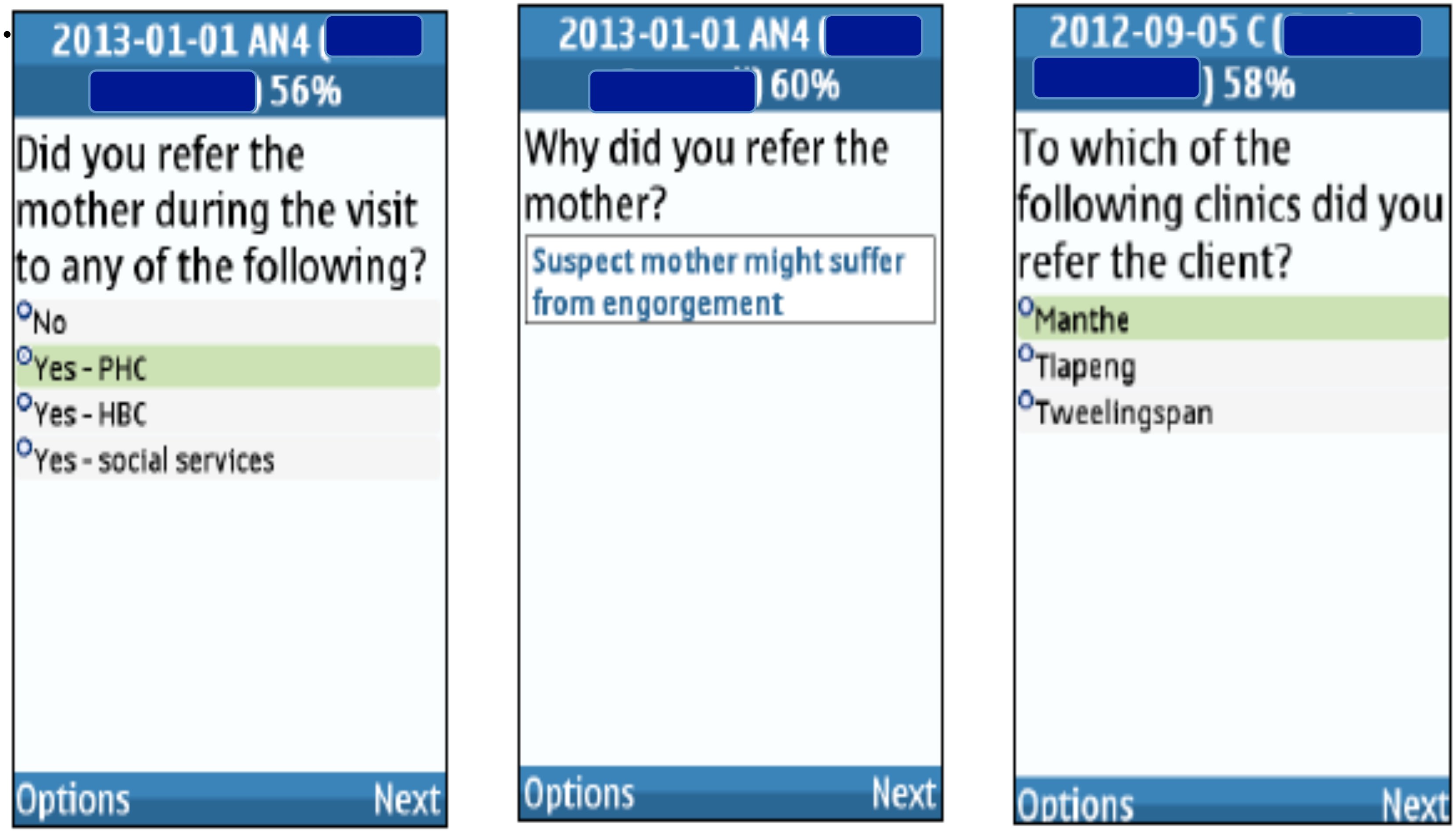

2.

Admin Main Console

Logged in as Mary Hartley Project : NW PHC Logout

health
Department: Health
REPUBLIC OF SOUTH AFRICA

DASHBOARD | CHW ACTIVITY | CHW HOUSEHOLD VISITS | DHIS DATA | HOUSEHOLDS | REFERRALS

Referrals

Select a Facility	CHW Name	Referral Status	Acknowledged	Referral From	Referral To	Outcome From	Outcome To
Manthe	All	All	All				

Search

CHW	Client	Referral Date	Referral Reason	Referral Status	Acknowledged	Outcome Date	Outcome Notes
[redacted]	[redacted]	2012-08-15	She has swollen gums	Expired	Yes		
[redacted]	[redacted]	2012-08-15	Check for CD4	Taken Up	Yes	2012-08-22	Cd4 checked and to come after 3 /12.
[redacted]	[redacted]	2012-08-17	For zentel VitaminA	Taken Up	Yes	2012-08-20	Deworming & vitamin A given, make follow up
[redacted]	[redacted]	2012-08-17	For VITAMIN A AND ZETEL	Taken Up	Yes	2012-08-20	Deworming & vit a given to the child, make follow up.
[redacted]	[redacted]	2012-08-20	For papsmear	Taken Up	Yes	2012-08-23	Client came in to do PAP smear, same performed To collect HPT & Epileptic treatment every month end.
[redacted]	[redacted]	2012-08-20	For immunisation for 2yrs ...	Taken Up	Yes	2012-08-22	Zentel given, vitamin A out of stock. To come monthly to check for vitamin A, then every 6 months up ...
[redacted]	[redacted]	2012-08-20	For Bp recheked	Expired	Yes		
[redacted]	[redacted]	2012-08-21	For checking weight and ...	Expired	Yes		
[redacted]	[redacted]	2012-08-21	To take treatment of high ...	Taken Up	Yes	2012-08-22	Client given ht rx.
[redacted]	[redacted]	2012-08-22	For papsmear	Taken Up	Yes	2012-09-03	Pap smear done & to come for results after six weeks.
[redacted]	[redacted]	2012-08-22	He missed immunization	Taken Up	Yes	2012-08-22	Vitamin A out of stock, to come monthly to check for vitamin A.
